# Supplementary material for: Efficacy of the PRESERFLO MicroShunt and a Meta-Analysis of the Literature
Source: J Clin Med. 2022 Dec 1;11(23):7149. doi: 10.3390/jcm11237149 (PMC9738249; doi:10.3390/jcm11237149)
Supplement: Supplementary file 1 [file jcm-11-07149-s001.zip › jcm-1999714-supplementary.pdf]

Supporting information

Efficacy of the PRESERFLO MicroShunt and a Meta-Analysis of the Literature

Contents:

Table S1: Summary of the retrieved studies from the systematic review.

Table S2: Quality assessment of the included studies according to the Newcastle–Ottawa Scale.

Figure S1: Scatterplot showing preoperative and postoperative IOP for PRESERFLO MicroShunt as a standalone procedure and as a cataract-combined procedure.

Figure S2: Flowchart showing the selection process for inclusion of studies from our searches according to the PRISMA guidelines.

Figure S3: Meta-analysis of the effect of the PRESERFLOMicroShunt on IOP for all specified time intervals.

Figure S4: Meta-analysis of the effect of the PRESERFLO MicroShunt on IOP-lowering medication for all specified time intervals.

Table S1. Summary of the retrieved studies from the systematic review

	STUDY		DESIGN	EYES PER GROUP (N)	DIAGNOSIS	INTERVENTION*	FOLLOW-UP (MONTHS)	IOP REDUCTION MMHG MEAN±SD (%)	NEEDLING (%)	SECONDARY SURGERIES (%)	HYPOTONY (%)	CHOROIDAL DETACHMENT/ EFFUSION (%)
**1**	Ahmed et al. 2022 [17]	R	Case series	8	POAG	PRESERFLO+ MMC	72	(30.7)	63	NA	NA	NA
**2**	Batlle et al. 2016 [18]	P	Observational study	23	POAG	PRESERFLO+MMC *(+CE n=9)*	36	(55)	NA	4.3	13	8.7
**3**	Beckers et al. 2021 [19]	P	Single arm Multicenter study	81	POAG	PRESERFLO+MMC	24	7.8±4.1	6.2	6.4	11.1	NA
**4**	Durr et al. 2022 [20]	R	Case series	85	Glaucoma	PRESERFLO+MMC	12	(43.3)	11.8	14.1	5.9	12.9
**5**	Baker et al. 2021 [28]	P	Randomized, Multicenter study	395	POAG	PRESERFLO+MMC	24	(29.1)	NA	NA	7.1	4.6
**6**	Barberá et al. 2022 [30]	R	Single-center, non-randomized	64	POAG	PRESERFLO+MMC (+CE=13)	9	(43.05)	1.6	15.6	7.8	7.8
**7**	Saletta et al. 2022 [29]	R	Chart review	30	Glaucoma	PRESERFLO+MMC	12	(34.7)	0.1	0.1	NA	NA
**8**	Tanner et al. 2022 [27]	R	Multicenter, Cohort study	104	Glaucoma	PRESERFLO+MMC	12	(37.2)	12.5	11.5	20.2	10.6
**9**	Fea et al. 2021 [21]	R	Open label, Multicenter study	104	Glaucoma	PRESERFLO+MMC	12	(43.8)	18.3	13.5	NA	4.8
**10**	Martinez -de la casa et. Al. 2021 [9]	R	Open label, Multicenter	58	Glaucoma	PRESERFLO+MMC *(+CE n=23)*	12	(32.1)	NA	NA	1.7	3.5
**11**	Quaranta et al. 2021 [22]	R	Chart review	31	POAG	PRESERFLO+MMC	12	(47.9)	19.3	3.3	19.3	9.6
**12**	Schlenker et al. 2020 [23]	R	Single center, Interventional, Case series	164	Glaucoma	PRESERFLO+MMC	12	(37.9)	8.5	1.8	1.2	9.2
**13**	Scheres et al. 2021 [24]	R	Comparative case series	41	POAG	PRESERFLO+MMC	24	(39)	5	22	39	2
**14**	Vastardis et al. 2021 [26]	R	Case series	25	POAG	PRESERFLO+MMC	6	(49.1)	NA	4	NA	NA

*= between brackets the number of eyes from the total study population that underwent a combined procedure, P=prospective, R=retrospective, CE=cataract extraction, MMC=mitomycin C, POAG=primary open angle glaucoma, SD= standard deviation, glaucoma= mixture of different types of glaucoma, NA=not applicable

Table S2. Quality assessment of the included non-randomized studies according to the Newcastle–Ottawa Scale.

	Author	*Selection*	*Comparability*	*Outcome (Cohort)/ Exposure Case control*	*Total score (max. 9)*
1	Ahmed et al. 2022 [17]	3	2	2	7
2	Batlle et al. 2016 [18]	3	2	3	8
3	Beckers et al. 2021 [19]	3	2	3	8
4	Durr et al. 2022 [20]	3	2	2	7
5	Baker et. al 2021 [28]	3	2	3	8
6	Barberá et al 2022 [30]	3	2	3	8
7	Saletta et al. 2022 [29]	3	2	3	8
8	Tanner et al. 2022 [27]	3	2	3	8
9	Fea et al. 2021 [21]	3	2	2	7
10	Martinez-de la casa et. al. 2021 [9]	3	2	3	8
11	Quaranta et al. 2021 [22]	3	2	2	7
12	Schlenker et al. 2020 [23]	3	2	3	8
13	Scheres et al. 2021 [24]	3	2	3	8
14	Vastardis et al. 2021 [26]	3	2	3	8

Figure S1. Scatterplot showing preoperative and postoperative IOP for PRESERFLO MicroShunt as a standalone and as a cataract-combined procedure.

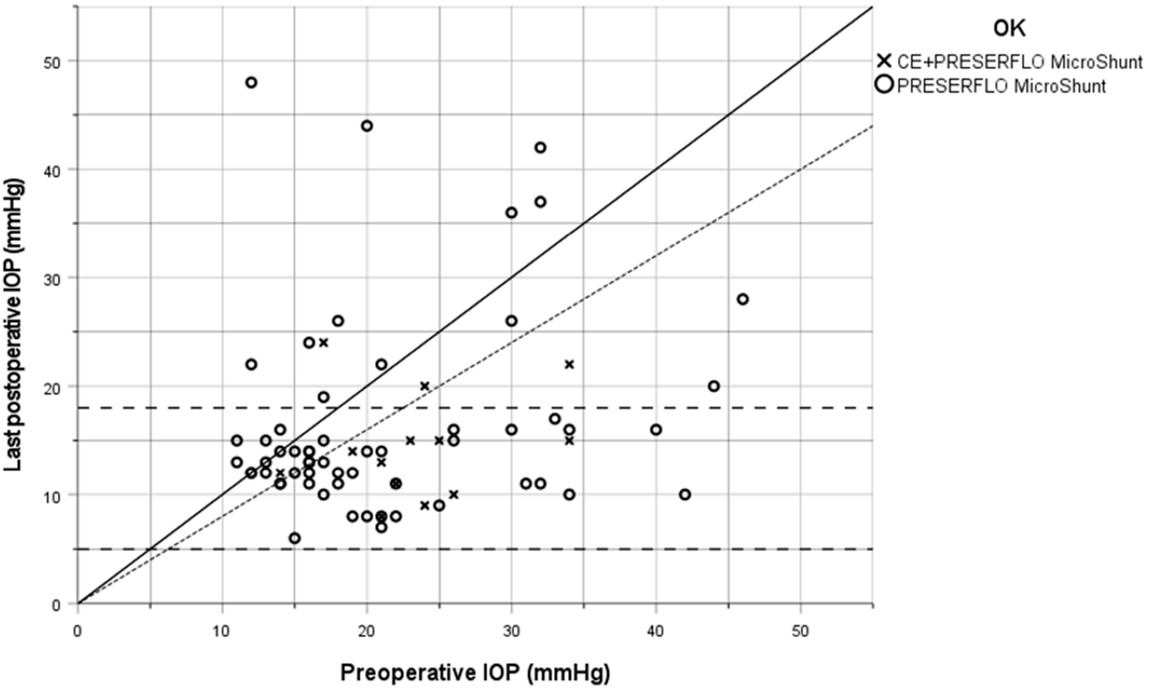

The dotted horizontal lines represent the 5mmHg and 18mmHg IOP-levels and the diagonal dotted line represents the 20% reduction level. IOP=intraocular pressure, CE= cataract extraction, PRESERFLO MicroShunt=standalone procedure, CE+PRESERFLO MicroShunt=combined procedure.

Figure S2. Flowchart showing the selection process for the inclusion and exclusion of the searched articles (according to PRISMA). PRISMA = Preferred Reporting Items for Systematic Reviews and Meta-Analyses.

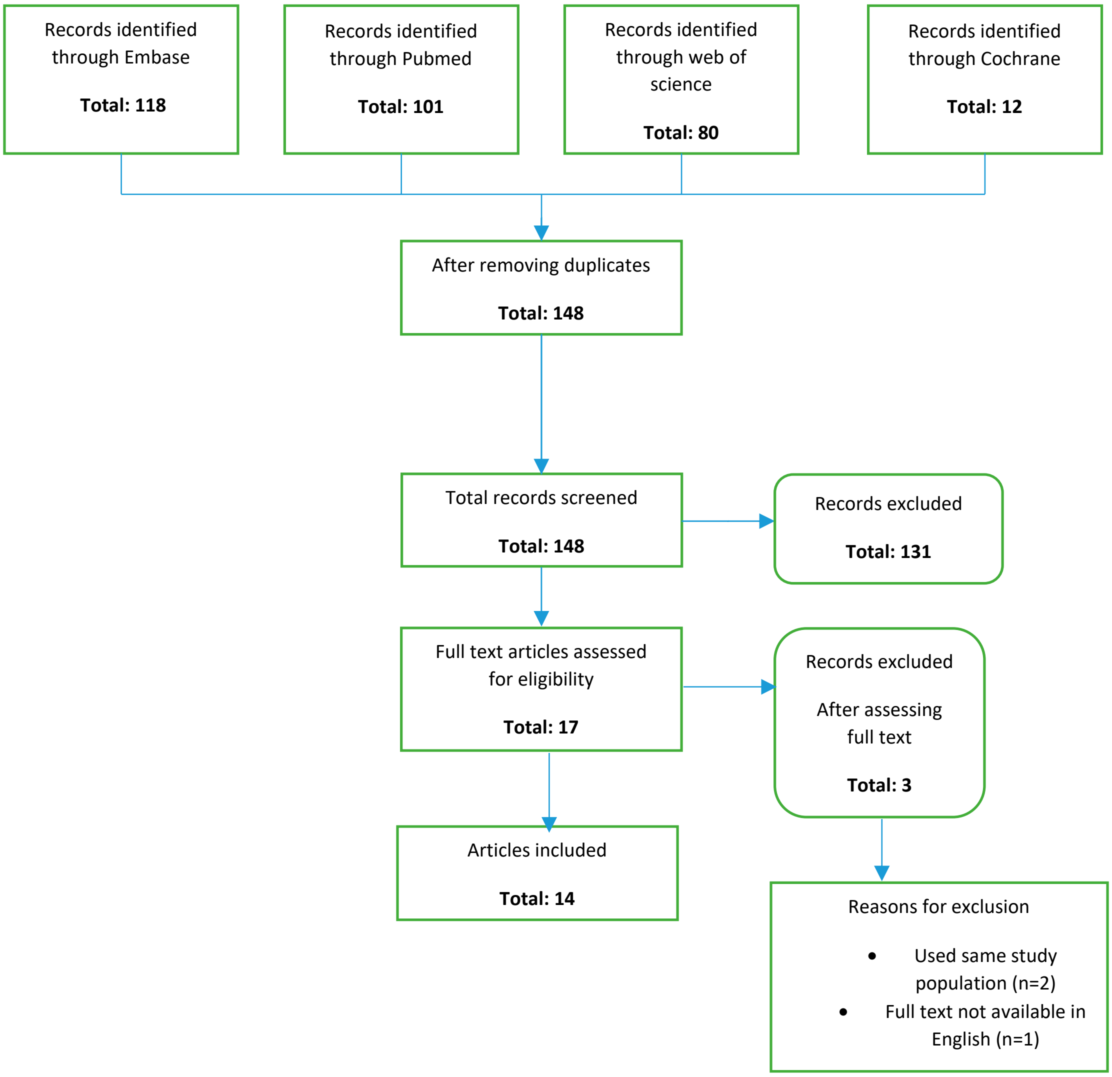

Search strings**: Embase.com** ('innfocus microshunt'/de OR ((styrene/de OR isobutylene/de OR 'poly(styrene block isobutylene block styrene)'/de) AND (glaucoma/exp OR 'glaucoma surgery'/exp OR 'intraocular pressure'/de OR 'glaucoma drainage implant'/de)) OR (Innfocus OR Preserflo OR (Aqueous-Humor NEAR/3 Shunt*) OR ((SIBS* OR Styrene-block-IsoButylene* OR polyIsoButylene* OR poly-IsoButylene* OR Styrene-IsoButylene*) NEAR/10 (glaucoma* OR subconjunctiv*-drain* OR intraocul* OR intra-ocul* OR microshunt* OR micro-shunt* OR ophthalmol*)) OR ((subconjunctiv* OR sub-conjunctiv*) NEAR/3 ab-externo NEAR/3 glaucoma) OR ((microshunt* OR micro-shunt*) NEAR/3 (ab-externo*))):ab,ti). **Medline All Ovid (Pubmed)** (((Styrene/ OR Styrenes/) AND (exp Glaucoma/ OR Intraocular Pressure/ OR Glaucoma Drainage Implants/)) OR (Innfocus OR Preserflo OR (Aqueous-Humor ADJ3 Shunt*) OR ((SIBS* OR Styrene-block-IsoButylene* OR polyIsoButylene* OR poly-IsoButylene* OR Styrene-IsoButylene*) ADJ10 (glaucoma* OR subconjunctiv*-drain* OR intraocul* OR intra-ocul* OR microshunt* OR micro-shunt* OR ophthalmol*)) OR ((subconjunctiv* OR sub-conjunctiv*) ADJ3 ab-externo ADJ3 glaucoma) OR ((microshunt* OR micro-shunt*) ADJ3 (ab-externo*))).ab,ti.) **Web of science (SCI-EXPANDED & SSCI, 1975-)** TS=(Innfocus OR Preserflo OR (Aqueous-Humor NEAR/2 Shunt*) OR ((SIBS* OR Styrene-block-IsoButylene* OR polyIsoButylene* OR poly-IsoButylene* OR Styrene-IsoButylene*) NEAR/10 (glaucoma* OR subconjunctiv*-drain* OR intraocul* OR intra-ocul* OR microshunt* OR micro-shunt* OR ophthalmol*)) OR ((subconjunctiv* OR sub-conjunctiv*) NEAR/2 ab-externo NEAR/2 glaucoma) OR ((microshunt* OR micro-shunt*) NEAR/2 (ab-externo**))) Cochrane Central Register of Controlled Trials** ((Innfocus OR Preserflo OR (Aqueous NEXT Humor NEAR/3 Shunt*) OR ((SIBS* OR Styrene NEXT block NEXT IsoButylene* OR polyIsoButylene* OR poly NEXT IsoButylene* OR Styrene NEXT IsoButylene*) NEAR/10 (glaucoma* OR subconjunctiv* NEXT drain* OR intraocul* OR intra NEXT ocul* OR microshunt* OR micro NEXT shunt* OR ophthalmol*)) OR ((subconjunctiv* OR sub NEXT conjunctiv*) NEAR/3 ab NEXT externo NEAR/3 glaucoma) OR ((microshunt* OR micro NEXT shunt*) NEAR/3 (ab NEXT externo*))):ab,ti)

Figure S3. Meta-analyses for the effect of the PRESERFLO MicroShunt on IOP for all specified time intervals.*

(A)

	pre-surgery			post-surgery 1d				Mean Difference	
**Study or Subgroup**	**Mean**	**SD**	**Total**	**Mean**	**SD**	**Total**	**Weight**	**IV, Fixed, 95% CI**	**Year**
Quaranta et al. [22]	24.12	3.14	31	7.58	1.8	31	1.6%	16.54 [15.27, 17.81]	2021
Scheres et al. [24]	20.1	5	41	7.9	4	41	0.7%	12.20 [10.24, 14.16]	2021
Vastardis et al. [26]	23.52	5.88	25	7.24	3.47	25	0.4%	16.28 [13.60, 18.96]	2021
Durr et al. [20]	23.8	7.9	85	7.8	5.1	85	0.7%	16.00 [14.00, 18.00]	2022
Saletta et al. [29]	23.6	8.3	30	8.9	4.4	30	0.2%	14.70 [11.34, 18.06]	2022
Tanner et al. [27]	23.4	0.8	104	7.1	0.3	90	95.4%	16.30 [16.13, 16.47]	2022
Barberá et al. [30]	22	6.3	64	8.3	0.3	64	1.1%	13.70 [12.15, 15.25]	2022
**Total (95% CI)**			**380**			**366**	**100.0%**	**16.24 [16.08, 16.40]**	

Heterogeneity: $Chi^2 = 28.28$, df = 6 (P < 0.0001); $I^2 = 79\%$
Test for overall effect: Z = 196.65 (P < 0.00001)

Mean Difference
IV, Fixed, 95% CI

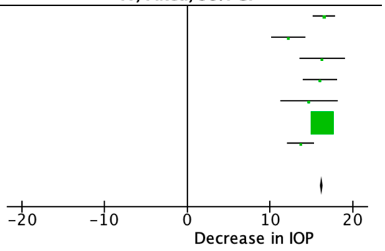

(B)

	Pre-surgery			Post surgery 1w				Mean Difference	
**Study or Subgroup**	**Mean**	**SD**	**Total**	**Mean**	**SD**	**Total**	**Weight**	**IV, Fixed, 95% CI**	**Year**
Scheres et al. [24]	20.1	5	41	8.7	4.1	41	0.7%	11.40 [9.42, 13.38]	2021
Vastardis et al. [26]	23.52	5.88	25	8.88	3.08	25	0.4%	14.64 [12.04, 17.24]	2021
Martinez et al. [9]	21.5	3.3	58	10.4	3.1	58	2.1%	11.10 [9.93, 12.27]	2021
Quaranta et al. [22]	24.12	3.14	31	8.22	2.82	31	1.3%	15.90 [14.41, 17.39]	2021
Tanner et al. [27]	23.4	0.8	104	9.1	0.4	98	93.6%	14.30 [14.13, 14.47]	2022
Durr et al. [20]	23.8	7.9	85	8.6	6	72	0.6%	15.20 [13.02, 17.38]	2022
Barberá et al. [30]	22	6.3	64	8.9	0.3	64	1.2%	13.10 [11.55, 14.65]	2022
Saletta et al. [29]	23.6	8.3	30	11.4	7	30	0.2%	12.20 [8.31, 16.09]	2022
**Total (95% CI)**			**438**			**419**	**100.0%**	**14.22 [14.06, 14.39]**	

Heterogeneity: $Chi^2 = 45.01$, df = 7 (P < 0.00001); $I^2 = 84\%$
Test for overall effect: Z = 166.60 (P < 0.00001)

Mean Difference
IV, Fixed, 95% CI

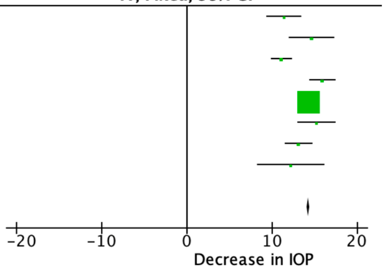

(C)

	Pre-surgery			Post-surgery 1m				Mean Difference	
**Study or Subgroup**	**Mean**	**SD**	**Total**	**Mean**	**SD**	**Total**	**Weight**	**IV, Fixed, 95% CI**	**Year**
Martinez et al. [9]	21.5	3.3	58	11.6	2.5	58	3.3%	9.90 [8.83, 10.97]	2021
Quaranta et al. [22]	24.12	3.14	31	10.19	2.6	31	1.8%	13.93 [12.49, 15.37]	2021
Scheres et al. [24]	20.1	5	41	10.3	3.2	40	1.1%	9.80 [7.98, 11.62]	2021
Vastardis et al. [26]	23.52	5.88	25	9.92	3.25	25	0.5%	13.60 [10.97, 16.23]	2021
Barberá et al. [30]	22	6.3	64	10.6	0.4	64	1.6%	11.40 [9.85, 12.95]	2022
Durr et al. [20]	23.8	7.9	85	10.1	4.7	69	0.9%	13.70 [11.69, 15.71]	2022
Saletta et al. [29]	23.6	8.3	30	13	6.8	30	0.3%	10.60 [6.76, 14.44]	2022
Tanner et al. [27]	23.4	0.8	104	13.1	0.7	102	90.3%	10.30 [10.09, 10.51]	2022
**Total (95% CI)**			**438**			**419**	**100.0%**	**10.42 [10.22, 10.61]**	

Heterogeneity: $Chi^2 = 43.01$, df = 7 (P < 0.00001); $I^2 = 84\%$
Test for overall effect: Z = 104.70 (P < 0.00001)

Mean Difference
IV, Fixed, 95% CI

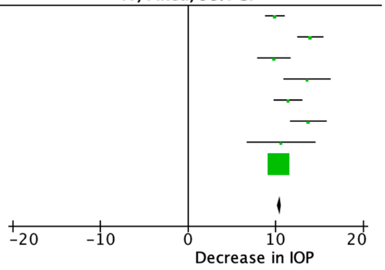

(D)

	Pre-surgery			Post-surgery 3m				Mean Difference	
**Study or Subgroup**	**Mean**	**SD**	**Total**	**Mean**	**SD**	**Total**	**Weight**	**IV, Fixed, 95% CI**	**Year**
Martinez et al. [9]	21.5	3.3	58	13.3	3.3	58	2.2%	8.20 [7.00, 9.40]	2021
Quaranta et al. [22]	24.12	3.14	31	11.12	3.93	31	1.0%	13.00 [11.23, 14.77]	2021
Scheres et al. [24]	20.1	5	41	10.9	2.8	38	1.0%	9.20 [7.43, 10.97]	2021
Vastardis et al. [26]	23.52	5.88	25	11.2	2.71	25	0.5%	12.32 [9.78, 14.86]	2021
Tanner et al. [27]	23.4	0.8	104	13.2	0.5	97	93.1%	10.20 [10.02, 10.38]	2022
Durr et al. [20]	23.8	7.9	85	11.3	5.3	67	0.7%	12.50 [10.39, 14.61]	2022
Barberá et al. [30]	22	6.3	64	11.1	0.3	64	1.3%	10.90 [9.35, 12.45]	2022
Saletta et al. [29]	23.6	8.3	30	16.6	6.7	30	0.2%	7.00 [3.18, 10.82]	2022
**Total (95% CI)**			**438**			**410**	**100.0%**	**10.20 [10.03, 10.38]**	

Heterogeneity: $Chi^2 = 32.24$, df = 7 (P < 0.0001); $I^2 = 78\%$
Test for overall effect: Z = 113.15 (P < 0.00001)

Mean Difference
IV, Fixed, 95% CI

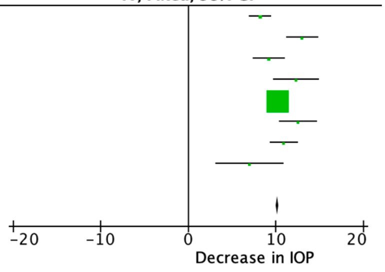

(E)

Study or Subgroup	Pre-surgery Mean	Pre-surgery SD	Pre-surgery Total	Post-surgery 6m Mean	Post-surgery 6m SD	Post-surgery 6m Total	Weight	Mean Difference IV, Fixed, 95% CI	Year
Martinez et al. [9]	21.5	3.3	58	14.3	3	55	2.7%	7.20 [6.04, 8.36]	2021
Quaranta et al. [22]	24.12	3.14	31	11.8	3.41	31	1.4%	12.32 [10.69, 13.95]	2021
Scheres et al. [24]	20.1	5	41	12.5	4.2	34	0.8%	7.60 [5.52, 9.68]	2021
Vastardis et al. [26]	23.52	5.88	25	12	3.13	25	0.5%	11.52 [8.91, 14.13]	2021
Durr et al. [20]	23.8	7.9	85	13	5.5	66	0.8%	10.80 [8.66, 12.94]	2022
Barberá et al. [30]	22	6.3	64	12.3	0.4	64	1.5%	9.70 [8.15, 11.25]	2022
Saletta et al. [29]	23.6	8.3	30	15.9	6.4	30	0.3%	7.70 [3.95, 11.45]	2022
Tanner et al. [27]	23.4	0.8	104	14.3	0.6	85	91.9%	9.10 [8.90, 9.30]	2022
**Total (95% CI)**			**438**			**390**	**100.0%**	**9.11 [8.92, 9.30]**	
Heterogeneity: Chi² = 34.05, df = 7 (P < 0.0001); I² = 79%									
Test for overall effect: Z = 93.24 (P < 0.00001)									

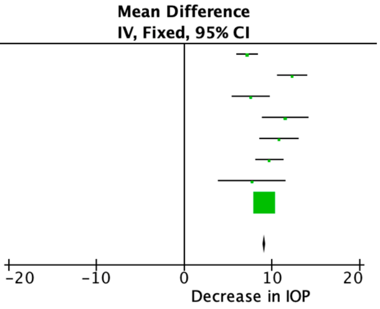

(F)

Study or Subgroup	Pre-surgery Mean	Pre-surgery SD	Pre-surgery Total	Post-surgery 1y Mean	Post-surgery 1y SD	Post-surgery 1y Total	Weight	Mean Difference IV, Fixed, 95% CI	Year
Batlle et al. [18]	23.8	5.3	23	10.7	2.8	23	0.5%	13.10 [10.65, 15.55]	2016
Quaranta et al. [22]	24.12	3.14	31	12.56	2.64	31	1.5%	11.56 [10.12, 13.00]	2021
Scheres et al. [24]	20.1	5	41	12.1	3.5	28	0.8%	8.00 [5.99, 10.01]	2021
Baker et al. [28]	21.1	4.9	395	14.3	4.3	332	6.9%	6.80 [6.13, 7.47]	2021
Beckers et al. [19]	21.7	3.4	81	14.5	4.6	67	1.8%	7.20 [5.87, 8.53]	2021
Fea et al. [21]	25.1	6.5	104	14.1	3.4	94	1.5%	11.00 [9.57, 12.43]	2021
Martinez et al. [9]	21.5	3.3	58	14.6	3.5	53	1.9%	6.90 [5.63, 8.17]	2021
Tanner et al. [27]	23.4	0.8	104	14.7	0.6	104	83.9%	8.70 [8.51, 8.89]	2022
Durr et al. [20]	23.8	7.9	85	13.5	5.4	76	0.7%	10.30 [8.23, 12.37]	2022
Ahmed et al. [17]	17.9	3.5	8	13.8	2.9	8	0.3%	4.10 [0.95, 7.25]	2022
Saletta et al. [29]	23.6	8.3	30	15.4	6.7	30	0.2%	8.20 [4.38, 12.02]	2022
**Total (95% CI)**			**960**			**846**	**100.0%**	**8.60 [8.42, 8.77]**	
Heterogeneity: Chi² = 90.83, df = 10 (P < 0.00001); I² = 89%									
Test for overall effect: Z = 95.76 (P < 0.00001)									

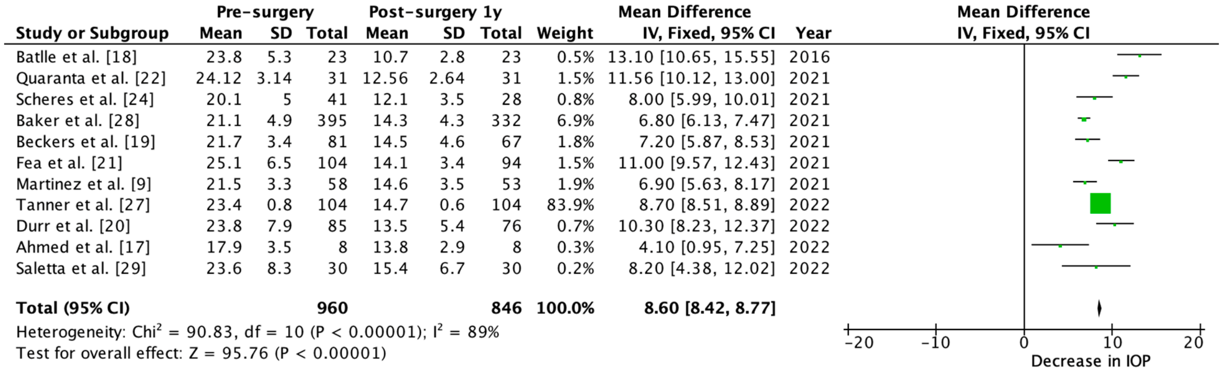

(H)

Study or Subgroup	Pre-surgery Mean	Pre-surgery SD	Pre-surgery Total	Post-surgery 2y Mean	Post-surgery 2y SD	Post-surgery 2y Total	Weight	Mean Difference IV, Fixed, 95% CI
Ahmed et al. [17]	17.9	3.5	8	12.8	2.3	8	9.4%	5.10 [2.20, 8.00]
Batlle et al. [18]	23.8	5.3	23	11.9	3.7	22	11.2%	11.90 [9.24, 14.56]
Beckers et al. [19]	21.7	3.4	81	14.1	3.2	60	65.6%	7.60 [6.50, 8.70]
Scheres et al. [24]	20.1	5	41	12.1	3.5	14	13.8%	8.00 [5.61, 10.39]
**Total (95% CI)**			**153**			**104**	**100.0%**	**7.90 [7.01, 8.79]**
Heterogeneity: Chi² = 12.55, df = 3 (P = 0.006); I² = 76%								
Test for overall effect: Z = 17.42 (P < 0.00001)								

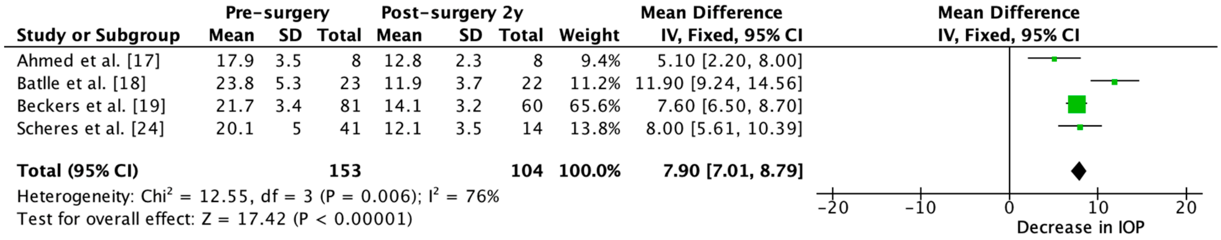

(I)

Study or Subgroup	Pre-surgery Mean	Pre-surgery SD	Pre-surgery Total	Post-surgery Mean	Post-surgery SD	Post-surgery Total	Weight	Mean Difference IV, Fixed, 95% CI
Ahmed et al. [17]	17.9	3.5	8	12.1	3.2	8	38.7%	5.80 [2.51, 9.09]
Batlle et al. [18]	23.8	5.3	23	10.7	3.5	22	61.3%	13.10 [10.49, 15.71]
**Total (95% CI)**			**31**			**30**	**100.0%**	**10.27 [8.23, 12.32]**
Heterogeneity: Chi² = 11.61, df = 1 (P = 0.0007); I² = 91%								
Test for overall effect: Z = 9.84 (P < 0.00001)								

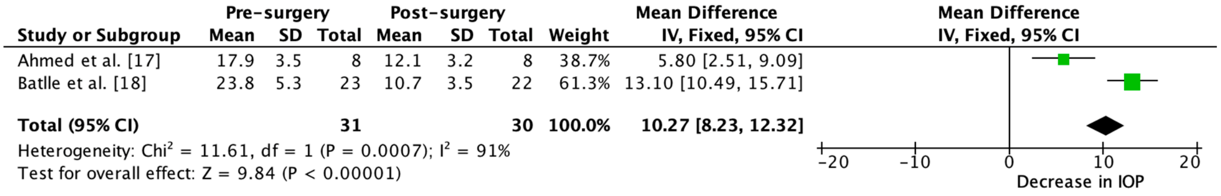

* = Presented as the mean decrease in IOP at 1 day (**A**), 1 week (**B**), 1month (**C**), 3 months (D), 6 months (**E**), 1 year (**F**), 2 years (**G**) and 3 years (**H**) follow-up. Black diamonds indicate the overall weighted mean difference (WMD). The size of the green box is inversely proportional to the variance. Horizontal lines indicate the 95% confidence interval (CI). The dashed vertical line in each panel shows the value for no effect (WMD = 0) compared to pre-surgery IOP. IOP = intraocular pressure; CI = confidence interval; SD = standard deviation; IV = inverse variance, WMD = weighted mean difference.

Figure S4. Meta-analysis for the effect of the PRESERFLO MicroShunt on IOP-lowering medication for all specified time-intervals.*

(A)

Study or Subgroup	Pre-surgery Mean	SD	Total	Post-surgery 1d Mean	SD	Total	Weight	Mean Difference IV, Fixed, 95% CI	Year
Quaranta et al. [22]	3.29	0.64	31	0	0	31		Not estimable	2021
Scheres et al. [24]	2.3	1.5	41	0.1	0.4	41	0.4%	2.20 [1.72, 2.68]	2021
Vastardis et al. [26]	2.52	0.91	25	0	0	25		Not estimable	2021
Durr et al. [20]	3.5	1.2	85	0	0	85		Not estimable	2022
Tanner et al. [27]	3.4	0.1	104	0.3	0.1	90	99.6%	3.10 [3.07, 3.13]	2022
**Total (95% CI)**			**286**			**272**	**100.0%**	**3.10 [3.07, 3.13]**	

Heterogeneity: $Chi^2 = 13.73$, df = 1 (P = 0.0002); $I^2 = 93\%$
Test for overall effect: Z = 215.49 (P < 0.00001)

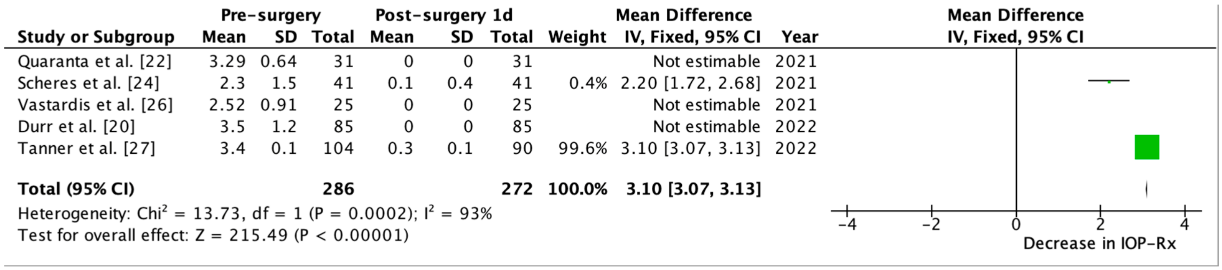

(B)

Study or Subgroup	Pre-surgery Mean	SD	Total	Post-surgery 1w Mean	SD	Total	Weight	Mean Difference IV, Fixed, 95% CI	Year
Quaranta et al. [22]	3.29	0.64	31	0	0	31		Not estimable	2021
Scheres et al. [24]	2.3	1.5	41	0	0.2	41	0.3%	2.30 [1.84, 2.76]	2021
Vastardis et al. [26]	2.52	0.91	25	0	0	25		Not estimable	2021
Durr et al. [20]	3.5	1.2	85	0	0.1	72	1.1%	3.50 [3.24, 3.76]	2022
Tanner et al. [27]	3.4	0.1	104	0.2	0.1	98	98.5%	3.20 [3.17, 3.23]	2022
**Total (95% CI)**			**286**			**267**	**100.0%**	**3.20 [3.17, 3.23]**	

Heterogeneity: $Chi^2 = 19.77$, df = 2 (P < 0.0001); $I^2 = 90\%$
Test for overall effect: Z = 229.04 (P < 0.00001)

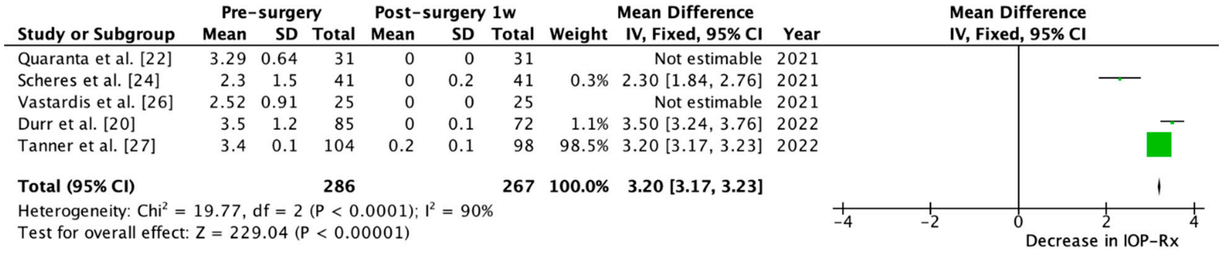

(C)

Study or Subgroup	Pre-surgery Mean	SD	Total	Post-surgery 1m Mean	SD	Total	Weight	Mean Difference IV, Fixed, 95% CI	Year
Scheres et al. [24]	2.3	1.5	41	0	0	40		Not estimable	2021
Vastardis et al. [26]	2.52	0.91	25	0	0	25		Not estimable	2021
Quaranta et al. [22]	3.29	0.64	31	0.06	0.24	31	1.3%	3.23 [2.99, 3.47]	2021
Tanner et al. [27]	3.4	0.1	104	0.3	0.1	102	97.8%	3.10 [3.07, 3.13]	2022
Durr et al. [20]	3.5	1.2	85	0.1	0.4	69	1.0%	3.40 [3.13, 3.67]	2022
**Total (95% CI)**			**286**			**267**	**100.0%**	**3.10 [3.08, 3.13]**	

Heterogeneity: $Chi^2 = 5.68$, df = 2 (P = 0.06); $I^2 = 65\%$
Test for overall effect: Z = 225.33 (P < 0.00001)

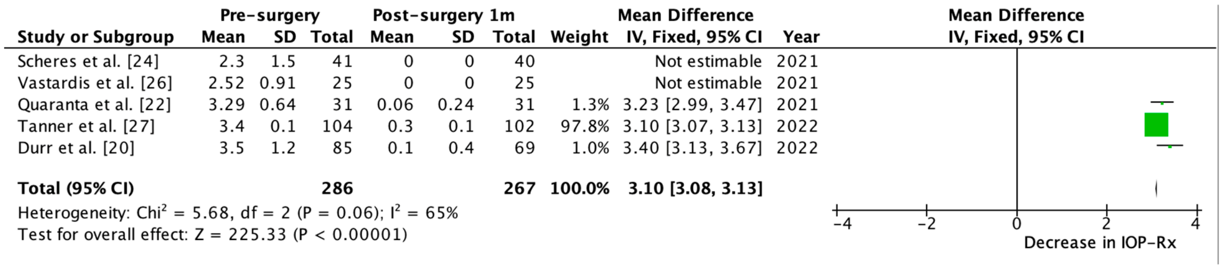

(D)

Study or Subgroup	Pre-surgery Mean	SD	Total	Post-surgery 3m Mean	SD	Total	Weight	Mean Difference IV, Fixed, 95% CI	Year
Quaranta et al. [22]	3.29	0.64	31	0.19	0.54	31	0.9%	3.10 [2.81, 3.39]	2021
Scheres et al. [24]	2.3	1.5	41	0.2	0.5	38	0.3%	2.10 [1.61, 2.59]	2021
Vastardis et al. [26]	2.52	0.91	25	0	0	25		Not estimable	2021
Tanner et al. [27]	3.4	0.1	104	0.5	0.1	97	97.9%	2.90 [2.87, 2.93]	2022
Durr et al. [20]	3.5	1.2	85	0.2	0.6	67	0.9%	3.30 [3.01, 3.59]	2022
**Total (95% CI)**			**286**			**258**	**100.0%**	**2.90 [2.88, 2.93]**	

Heterogeneity: $Chi^2 = 19.32$, df = 3 (P = 0.0002); $I^2 = 84\%$
Test for overall effect: Z = 207.78 (P < 0.00001)

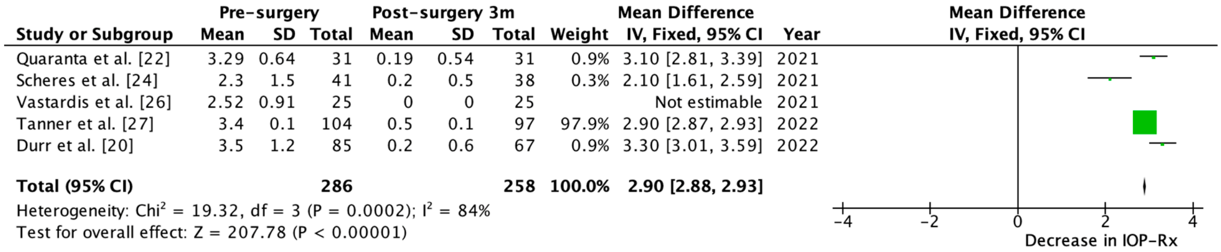

(E)

Study or Subgroup	Pre-surgery Mean	Pre-surgery SD	Pre-surgery Total	Post-surgery 6m Mean	Post-surgery 6m SD	Post-surgery 6m Total	Weight	Mean Difference IV, Fixed, 95% CI	Year
Quaranta et al. [22]	3.29	0.64	31	0.26	0.58	31	0.9%	3.03 [2.73, 3.33]	2021
Scheres et al. [24]	2.3	1.5	41	0.2	0.5	34	0.3%	2.10 [1.61, 2.59]	2021
Vastardis et al. [26]	2.52	0.91	25	0.04	0.2	25	0.6%	2.48 [2.11, 2.85]	2021
Tanner et al. [27]	3.4	0.1	104	0.6	0.1	85	97.5%	2.80 [2.77, 2.83]	2022
Durr et al. [20]	3.5	1.2	85	0.4	0.9	66	0.7%	3.10 [2.77, 3.43]	2022
**Total (95% CI)**			**286**			**241**	**100.0%**	**2.80 [2.77, 2.83]**	
Heterogeneity: $Chi^2 = 16.10$, df = 4 (P = 0.003); $I^2 = 75\%$									
Test for overall effect: Z = 193.94 (P < 0.00001)									

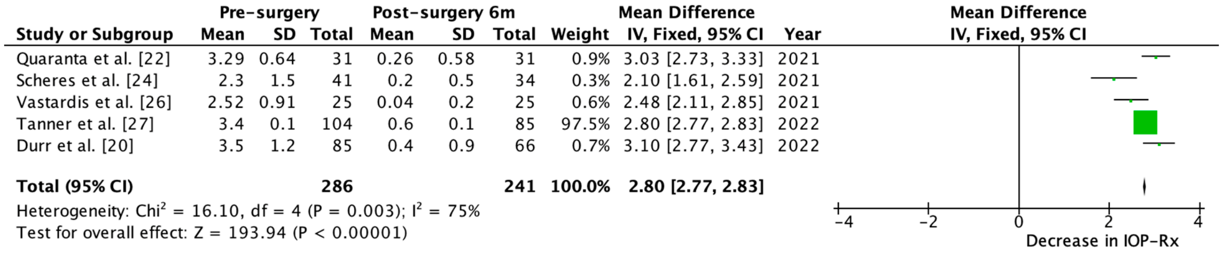

(F)

Study or Subgroup	Pre-surgery Mean	Pre-surgery SD	Pre-surgery Total	Post-surgery 1y Mean	Post-surgery 1y SD	Post-surgery 1y Total	Weight	Mean Difference IV, Fixed, 95% CI
Ahmed et al. [17]	3.5	0.5	8	1.6	1.4	8	0.1%	1.90 [0.87, 2.93]
Baker et al. [28]	3.1	1	395	0.6	1.1	384	3.1%	2.50 [2.35, 2.65]
Batlle et al. [18]	2.4	0.9	23	0.3	0.8	23	0.3%	2.10 [1.61, 2.59]
Durr et al. [20]	3.5	1.2	85	0.9	1.4	76	0.4%	2.60 [2.19, 3.01]
Fea et al. [21]	3	1	104	0.77	0.95	94	0.9%	2.23 [1.96, 2.50]
Martinez et al. [9]	2.3	0.5	58	0.2	0.5	53	2.0%	2.10 [1.91, 2.29]
Quaranta et al. [22]	3.29	0.64	31	0.46	0.77	31	0.5%	2.83 [2.48, 3.18]
Scheres et al. [24]	2.3	1.5	41	0.6	1	28	0.2%	1.70 [1.11, 2.29]
Tanner et al. [27]	3.4	0.1	104	0.7	0.1	104	92.5%	2.70 [2.67, 2.73]
**Total (95% CI)**			**849**			**801**	**100.0%**	**2.67 [2.65, 2.70]**
Heterogeneity: $Chi^2 = 74.35$, df = 8 (P < 0.00001); $I^2 = 89\%$								
Test for overall effect: Z = 200.50 (P < 0.00001)								

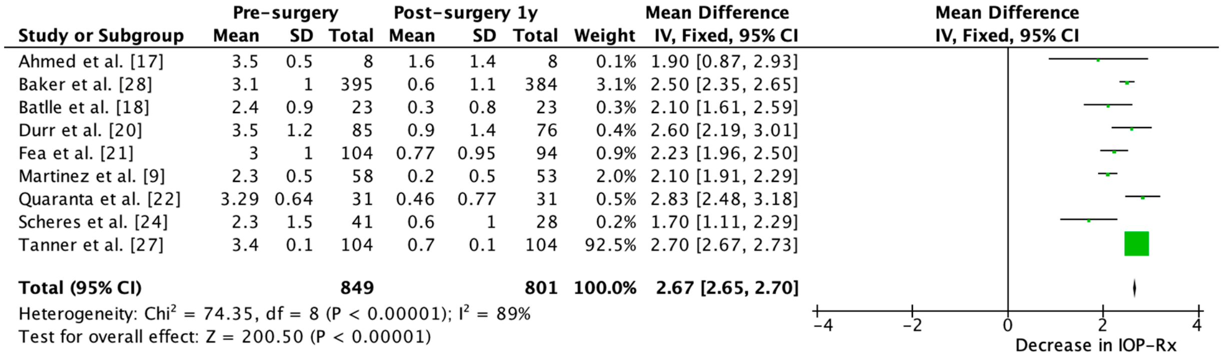

(G)

Study or Subgroup	Pre-surgery Mean	Pre-surgery SD	Pre-surgery Total	Post-surgery 2y Mean	Post-surgery 2y SD	Post-surgery 2y Total	Weight	Mean Difference IV, Fixed, 95% CI
Ahmed et al. [17]	3.5	0.5	8	1.6	1.6	8	5.5%	1.90 [0.74, 3.06]
Batlle et al. [18]	2.4	0.9	23	0.4	1	22	24.0%	2.00 [1.44, 2.56]
Beckers et al. [19]	2.1	1.3	81	0.5	0.9	61	56.8%	1.60 [1.24, 1.96]
Scheres et al. [24]	2.3	1.5	41	0.7	1.1	14	13.7%	1.60 [0.86, 2.34]
**Total (95% CI)**			**153**			**105**	**100.0%**	**1.71 [1.44, 1.99]**
Heterogeneity: $Chi^2 = 1.58$, df = 3 (P = 0.66); $I^2 = 0\%$								
Test for overall effect: Z = 12.30 (P < 0.00001)								

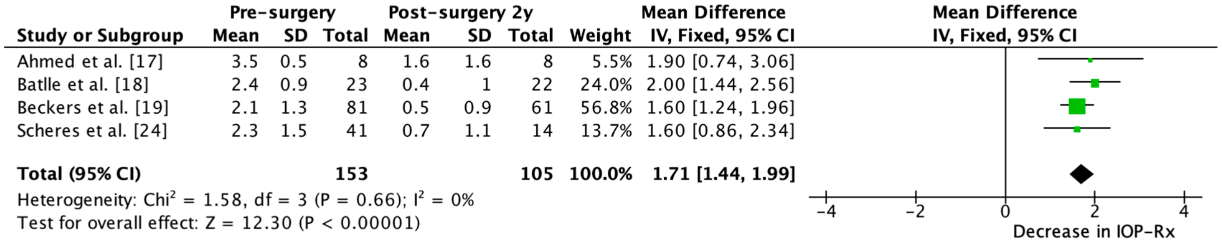

(H)

Study or Subgroup	Pre-surgery Mean	Pre-surgery SD	Pre-surgery Total	Post-surgery 3y Mean	Post-surgery 3y SD	Post-surgery 3y Total	Weight	Mean Difference IV, Fixed, 95% CI
Ahmed et al. [17]	3.5	0.5	8	1.5	1.4	8	24.6%	2.00 [0.97, 3.03]
Batlle et al. [18]	2.4	0.9	23	0.7	1.1	22	75.4%	1.70 [1.11, 2.29]
**Total (95% CI)**			**31**			**30**	**100.0%**	**1.77 [1.26, 2.28]**
Heterogeneity: $Chi^2 = 0.25$, df = 1 (P = 0.62); $I^2 = 0\%$								
Test for overall effect: Z = 6.80 (P < 0.00001)								

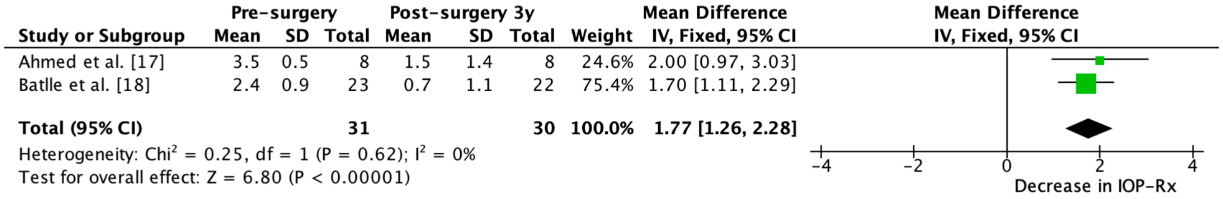

* = Presented as the mean decrease in IOP-lowering medication at 1 day (**A**), 1 week (**B**), 1 month (**C**), 3 months (**D**), 6 months (**E**), 1 year (**F**), 2 years (**G**), 3 years (**H**) of follow-up. Black diamonds indicate the overall weighted mean difference (WMD). The size of the green box is inversely proportional to the variance. Horizontal lines indicate 95% confidence interval (CI). The dashed vertical line in each panel shows the value for no effect (WMD = 0) compared to pre-surgery IOP-lowering medications. IOP = intraocular pressure; CI = confidence interval; SD = standard deviation; IV = inverse variance, WMD = weighted mean difference
